# Association between copy number variations in parkin (*PRKN*) and schizophrenia and autism spectrum disorder: A case–control study

**DOI:** 10.1002/npr2.12370

**Published:** 2023-11-01

**Authors:** Tzuyao Lo, Itaru Kushima, Hiroki Kimura, Branko Aleksic, Takashi Okada, Hidekazu Kato, Toshiya Inada, Yoshihiro Nawa, Youta Torii, Maeri Yamamoto, Ryo Kimura, Yasuko Funabiki, Hirotaka Kosaka, Shusuke Numata, Kiyoto Kasai, Tsukasa Sasaki, Shigeru Yokoyama, Toshio Munesue, Ryota Hashimoto, Yuka Yasuda, Michiko Fujimoto, Masahide Usami, Masanari Itokawa, Makoto Arai, Kazutaka Ohi, Toshiyuki Someya, Yuichiro Watanabe, Jun Egawa, Tsutomu Takahashi, Michio Suzuki, Hidenori Yamasue, Nakao Iwata, Masashi Ikeda, Norio Ozaki

**Affiliations:** ^1^ Department of Psychiatry Nagoya University Graduate School of Medicine Nagoya Japan; ^2^ Medical Genomics Center Nagoya University Hospital Nagoya Japan; ^3^ Department of Developmental Disorders, National Institute of Mental Health National Center of Neurology and Psychiatry Nagoya Japan; ^4^ Department of Anatomy and Developmental Biology Graduate School of Medicine Kyoto University Kyoto Japan; ^5^ Department of Cognitive, Behavioral and Health Sciences, Graduate School of Human and Environmental Studies Kyoto University Kyoto Japan; ^6^ Department of Neuropsychiatry, Faculty of Medical Sciences University of Fukui Fukui Japan; ^7^ Department of Psychiatry, Graduate School of Biomedical Science Tokushima University Tokushima Japan; ^8^ Department of Neuropsychiatry, Graduate School of Medicine University of Tokyo Tokyo Japan; ^9^ International Research Center for Neurointelligence at University of Tokyo Institutes for Advanced Study Tokyo Japan; ^10^ Laboratory of Health Education, Graduate School of Education University of Tokyo Tokyo Japan; ^11^ Research Center for Child Mental Development Kanazawa University Ishikawa Japan; ^12^ Department of Pathology of Mental Diseases National Institute of Mental Health National Center of Neurology and Psychiatry Tokyo Japan; ^13^ Department of Psychiatry Osaka University Graduate School of Medicine Osaka Japan; ^14^ Department of Child and Adolescent Psychiatry Kohnodai Hospital, National Center for Global Health and Medicine Chiba Japan; ^15^ Schizophrenia Research Project, Department of Psychiatry and Behavioral Sciences Tokyo Metropolitan Institute of Medical Science Tokyo Japan; ^16^ Department of Psychiatry Tokyo Metropolitan Matsuzawa Hospital Tokyo Japan; ^17^ Department of Psychiatry Gifu University Graduate School of Medicine Gifu Japan; ^18^ Department of General Internal Medicine Kanazawa Medical University Ishikawa Japan; ^19^ Department of Psychiatry Niigata University Graduate School of Medical and Dental Sciences Niigata Japan; ^20^ Department of Neuropsychiatry University of Toyama Graduate School of Medicine and Pharmaceutical Sciences Toyama Japan; ^21^ Research Center for Idling Brain Science University of Toyama Toyama Japan; ^22^ Department of Psychiatry Hamamatsu University School of Medicine Hamamatsu Japan; ^23^ Department of Psychiatry Fujita Health University School of Medicine Toyoake Japan; ^24^ Institute for Glyco‐core Research Nagoya University Nagoya Japan

**Keywords:** autism spectrum disorder, DNA copy number variations, parkin, Parkinson disease 2, schizophrenia

## Abstract

**Aim:**

The present study aimed to examine the association between copy number variations (CNVs) in parkin (*PRKN*) and schizophrenia (SCZ) and autism spectrum disorder (ASD) in a large case–control sample.

**Method:**

Array comparative genomic hybridization was performed on 3111 cases with SCZ, 1236 cases with ASD, and 2713 controls. We systematically prioritized likely pathogenic CNVs (LP‐CNVs) in *PRKN* and examined their association with SCZ and ASD.

**Results:**

In total, 3014 SCZ cases (96.9%), 1205 ASD cases (97.5%), and 2671 controls (98.5%) passed quality control. We found that monoallelic carriers of LP‐CNVs in *PRKN* were common (70/6890, 1.02%) and were not at higher risk of SCZ (*p* = 0.29) or ASD (*p* = 0.72). We observed that the distribution pattern of LP‐CNVs in the Japanese population was consistent with those in other populations. We also identified a patient diagnosed with SCZ and early‐onset Parkinson's disease carrying biallelic pathogenic CNVs in *PRKN*. The absence of Parkinson's symptoms in 10 other monoallelic carriers of the same pathogenic CNV further reflects the lack of effect of monoallelic pathogenic variants in *PRKN* in the absence of a second hit.

**Conclusion:**

The present findings suggest that monoallelic CNVs in *PRKN* do not confer a significant risk for SCZ or ASD. However, further studies to investigate the association between biallelic CNVs in *PRKN* and SCZ and ASD are warranted.

## Abstract 2

The study analyzed a large case–control sample of 3111 schizophrenia (SCZ) cases, 1236 autism spectrum disorder (ASD) cases, and 2713 controls to investigate the association between copy number variations (CNVs) in the parkin (*PRKN*) gene and neuropsychiatric disorders. The study found that monoallelic carriers of likely pathogenic CNVs in *PRKN* did not show a significant risk for SCZ or ASD. However, the study identified a patient with both SCZ and early‐onset Parkinson's disease who carries biallelic pathogenic CNVs in *PRKN*. This finding prompts further investigation into the relationship between biallelic CNVs in *PRKN* and the co‐occurrence of neuropsychiatric disorders.

## Graphical abstract

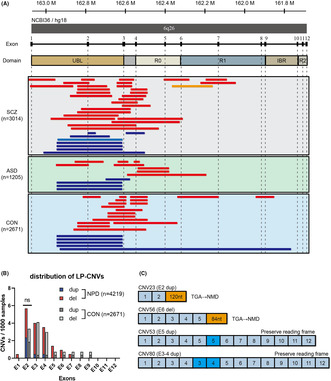

## 1 INTRODUCTION

**FIGURE 1:**
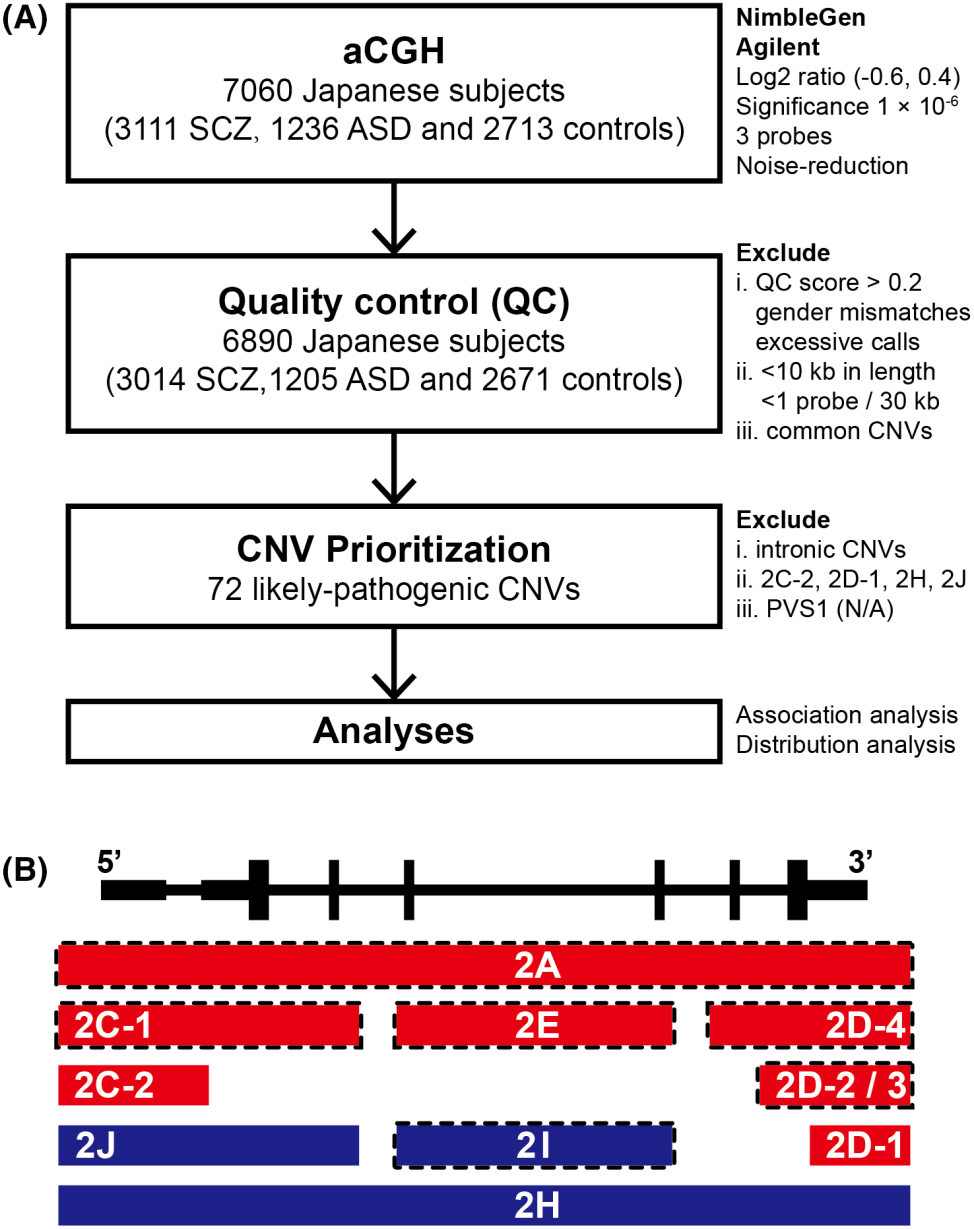
Workflow and classifications of CNVs in the present study. (A) Workflow of the present study. (B) Schematic illustration of classifications based on overlapping patterns following the guidelines for the interpretation of CNVs provided by ACMG and ClinGen. Red bars and dark blue bars represent deletions and duplications, respectively. Bars framed by the dotted line represent classifications prioritized as LP‐CNVs. 2A: Deletions completely overlapping an established HI gene/genomic region; 2C‐1: Deletions partially overlapping with the 5′ end (3′ end not involved) and the coding sequence is involved; 2C‐2: Deletions partially overlapping with the 5′ end (3′ end not involved) and only the 5’ UTR is involved; 2D‐1: Deletions partially overlapping with the 3′ end (5′ end not involved) and only the 3′ untranslated region is involved; 2D‐2/3: Deletions partially overlapping with the 3′ end (5′ end not involved) and only the last exon is involved; 2D‐4: Deletions partially overlapping with the 3′ end (5′ end not involved) and includes other exons in addition to the last exon. Nonsense‐mediated decay is expected to occur; 2E: Deletions with both breakpoints within the same gene; 2H: Duplications fully containing an HI gene; 2I: Duplications with both breakpoints within the same gene; 2 J: Duplications with one breakpoint within the established HI gene. ACMG, American College of Medical Genetics and Genomics; ClinGen, Clinical Genome Resource; HI, haploinsufficient; LP‐CNV, likely pathogenic copy number variation.

The encoding gene of parkin, *PRKN* (OMIM *602544), formerly known as *PARK2*, was originally discovered in 1998 by Kitada et al.^1^ and named after its role in the pathogenesis of autosomal recessive juvenile Parkinson disease‐2 (PARK2, MIM 600116). Soon thereafter, further works established the genetic variants of *PRKN* as a common cause of early‐onset Parkinson's disease (EOPD).^2^, ^3^ Since the original discovery of exonic deletions in *PRKN*, copy number variations (CNVs) have been the main focus of genetic studies in *PRKN* because they likely lead to functional loss of Parkin. As studies have indicated a substantial contribution of CNVs to the etiology of neuropsychiatric disorders (NPDs),^4^, ^5^, ^6^, ^7^ the identification of CNVs in *PRKN* among patients with schizophrenia (SCZ) and autism spectrum disorder (ASD) has brought the possible involvement of *PRKN* in NPDs to light^8^, ^9^ This is further supported by neurobiological evidence that knockdown of Parkin causes reduced surface levels of AMPA and NMDA receptors and alters glutamatergic synaptic transmission, which is a common feature of synaptopathy in SCZ and ASD.^10^, ^11^, ^12^ Thereafter, several reports of patients with NPDs carrying CNVs in *PRKN* were published,^13^, ^14^, ^15^ and case–control association studies attempted to clarify the role of heterozygous CNVs in *PRKN* in NPDs, including attention‐deficit/hyperactivity disorder (ADHD)^16^, ^17^ and ASD.^18^, ^19^ While these early approaches implicated a possible association of CNVs in *PRKN* with NPDs, the genotyping methods used in these studies may have missed some of the CNVs, and the results were inconsistent or lacked sufficient power because of their limited sample sizes (ranging from about 340 to 880^16^, ^17^, ^18^, ^19^). Accompanying the limited sample sizes was the lack of proper filtering. As CNVs are rare events, studies tend to adopt loose filtering to preserve more CNVs, resulting in the inclusion of not only likely pathogenic CNVs (e.g., CNVs at internal exons), but also less likely pathogenic CNVs^20^ (e.g., intronic CNVs,^8^, ^16^ duplications spanning terminal [first/last] exons) in the same study. These defects in study design may have led to inaccurate interpretations of the role of CNVs in *PRKN* in the pathogenesis of NPDs. Therefore, a case–control association study with an adequate sample size and appropriate prioritization using a sensitive and reliable detection method is needed to clarify the role of CNVs in *PRKN* in NPDs. To address this issue, we performed array comparative genomic hybridization (aCGH) on 4347 NPD cases (3111 SCZ and 1236 ASD) and 2713 healthy controls. We adopted a systematic prioritization for likely pathogenic CNVs (LP‐CNVs) (Figure 1) and examined the association between LP‐CNVs in *PRKN* and NPDs. Additionally, we analyzed the frequency and distribution of LP‐CNVs in *PRKN* in the Japanese population and compared our findings with previous reports in other populations. We reported a biallelic carrier of pathogenic CNVs in *PRKN* diagnosed with SCZ and EOPD, along with other monoallelic carries of the same pathogenic CNV to investigate the effect of monoallelic pathogenic variants in *PRKN* in the absence of a second hit.

## 2 METHODS

### 2.1 Participants

All participants recruited into the present study, including 3111 SCZ cases, 1236 ASD cases, and 2713 healthy controls, were of Japanese ancestry. SCZ and ASD were diagnosed based on the criteria set forth in the Diagnostic and Statistical Manual of Mental Disorders, Fifth Edition. The healthy controls were selected from the general population and confirmed to have no history of mental disorders based on self‐reported information and questionnaires. This study was approved by the ethics committee of the Nagoya University and affiliated institutes. Written informed consent was obtained from all participants or accompanying family members before the study began.

### 2.2 Array comparative genomic hybridization (aCGH)

Two aCGH platforms were utilized for CNV detection in this study: the NimbleGen 720 K Whole‐Genome Tiling array (Roche NimbleGen) and the Agilent SurePrint G3 human CGH 400 K (Agilent). CNV calls were generated using the Fast Adaptive States Segmentation Technique 2 algorithm implemented in Nexus Copy Number software v9.0 (BioDiscovery). The log_2_ ratio thresholds for CNV calls on both platforms were set as follows: (i) 10–500 kb: −0.6 (deletion) and 0.4 (duplication); and (ii) >500 kb: −0.4 (deletion) and 0.3 (duplication). A significance threshold of 1 × 10^−6^ was applied to adjust the sensitivity of the segmentation algorithm, and at least three contiguous probes were required for a CNV call in both the NimbleGen and Agilent arrays. To systematically correct for artifacts caused by GC content and fragment length, a noise‐reduction algorithm for aCGH data was adopted.^21^ The accuracy of CNVs identified by aCGH was confirmed in our previous study using a quantitative real‐time polymerase chain reaction (TaqMan copy number assays) (Applied Biosystems).^22^ For quality control (QC), a score was calculated for each sample based on the statistical variance of the probe‐to‐probe log ratios. Lower QC scores indicated higher quality results with less variation, whereas scores >0.2 were excluded from the analysis, as were samples with gender mismatches or excessive autosomal CNV calls. We then filtered out CNV calls that were < 10 kb in length or had low probe density (<1 probe/30 kb). Finally, we removed common CNVs that appeared in ≥1% of the total sample).

### 2.3 Prioritization of likely pathogenic CNVs in *PRKN*

We started by considering all CNVs in the *PRKN* locus (chr6: 161691121‐163 068 690, NCBI36) as potential candidates. To identify CNVs that were likely pathogenic, we performed a systematic prioritization process (Figure 1A). First, we excluded all intronic CNVs because they were deemed less likely to be pathogenic. Next, to prioritize the pathogenic CNVs that cause functional loss of one copy of *PRKN*, we followed the guidelines for interpreting CNVs provided by the American College of Medical Genetics and Genomics and the Clinical Genome Resource (Figure 1B),^23^ under the assumption of *PRKN* being a haploinsufficient gene, although this is still under debate. We excluded CNVs falling in categories 2C‐2, 2D‐1, 2H, and 2 J, which have insufficient evidence to support their pathogenicity, and retained CNVs falling in categories 2A, 2C‐1, 2D‐2, 2D‐3, 2D‐4, 2E, and 2I. We further evaluated the CNVs in categories 2E and 2I based on PVS1 specifications.^24^ 2E and 2I CNVs disrupting reading frames and predicted to undergo nonsense‐mediated decay (NMD) were classified as PVS1. 2E CNVs preserving reading frames were classified as PVS1_Strong (if removing >10% of protein) or PVS1_Moderate (if removing <10% of protein). 2I CNVs preserving reading frames were classified as N/A. CNVs classified as PVS1, PVS1_Strong, or PVS1_Moderate were retained, whereas those classified as N/A were excluded. After this prioritization process, we retained the CNVs considered to have moderate to very strong evidence supporting their pathogenicity and labeled these as likely pathogenic CNVs (LP‐CNVs; Table S1).

### 2.4 Association analysis

We conducted association analysis on carriers of monoallelic (heterozygous) and biallelic (homozygous or compound heterozygous) CNVs separately (Figure 1A). To investigate the associations between LP‐CNVs in *PRKN* and SCZ or ASD, we used Fisher's exact test (two‐tailed) to calculate odds ratios (ORs) and *P*‐values for the contingency table. In a secondary analysis, we investigated the association between LP‐CNVs that spanned specific exon(s) and SCZ or ASD using Fisher's exact test (two‐tailed).

## 3 RESULTS

### 3.1 Identification of *PRKN* CNVs by aCGH

**FIGURE 2:**
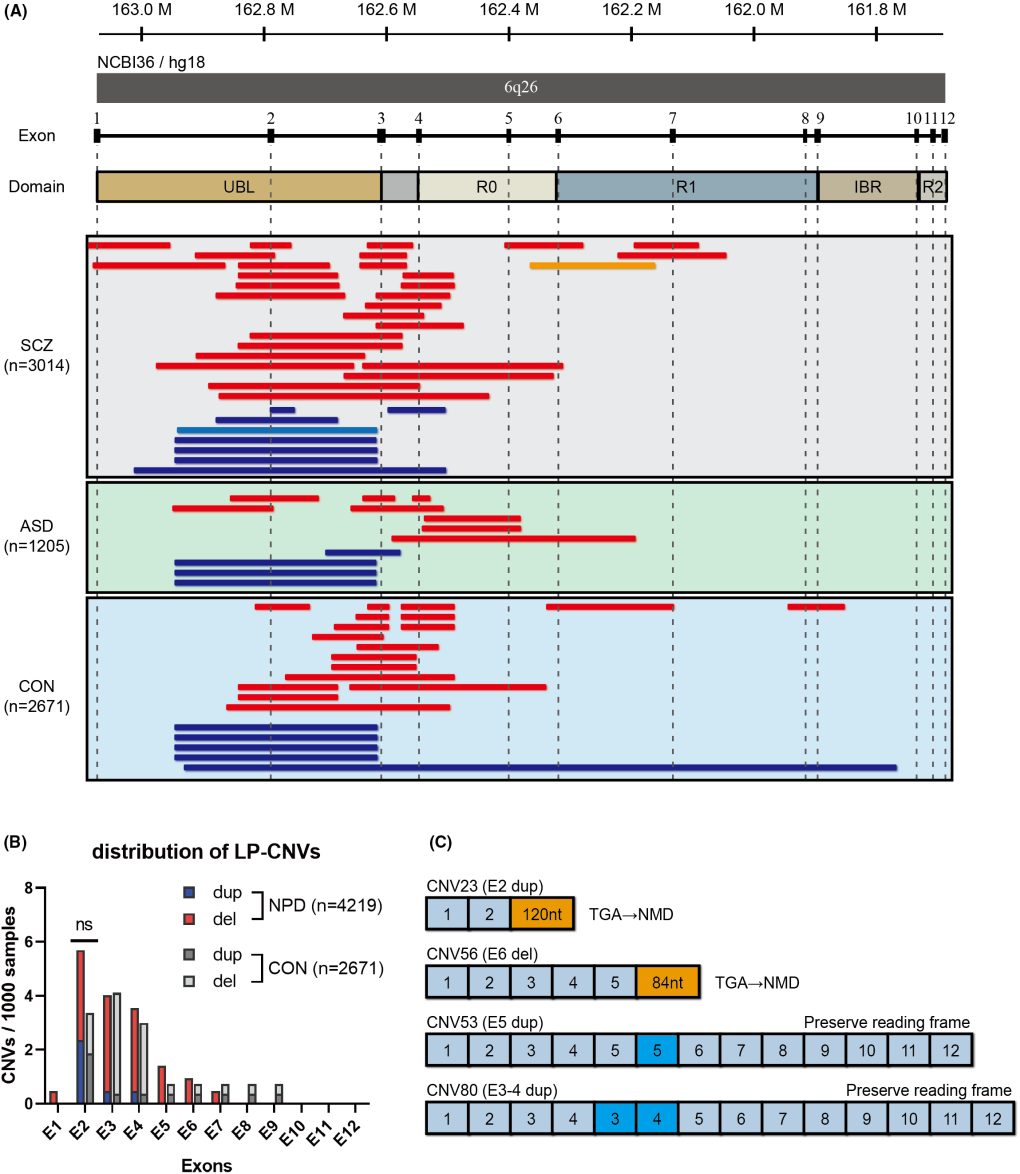
Genomic locations, distribution, and consequences of LP‐CNVs in *PRKN*. (A) Genomic locations of LP‐CNVs in *PRKN*. Red bars and dark blue bars represent deletions and duplications identified in the monoallelic carriers, respectively. The yellow bar and light blue bar represent the deletion (CNV56) and the duplication (CNV23) identified in the biallelic carrier. The genomic coordinates correspond to the NCBI36/hg18 build of the human genome assembly. (B) Distribution of LP‐CNVs in the 12 exons of *PRKN* in NPD cases (*n* = 4219) and controls (*n* = 2671). (C) Predicted consequences of the mRNA structure due to CNVs in *PRKN*. Duplication or deletion disrupting reading frames and resulting in a premature stop codon that leads to NMD (e.g., CNV23 and CNV56). Duplication spanning one or more exons preserving reading frames and leading to mRNA with exon repetition (e.g., CNV53, and CNV80). ASD, autism spectrum disorder; CON, healthy controls; LP‐CNV, likely pathogenic copy number variation; del, deletion; dup, duplication; NMD, nonsense‐mediated mRNA decay; NPD, neuropsychiatric disorder; nt, nucleotide; SCZ, schizophrenia.

Of the 7060 individuals (3111 SCZ cases, 1236 ASD cases, and 2713 controls) analyzed with aCGH, 3014 SCZ cases (96.9%), 1205 ASD cases (97.5%), and 2671 controls (98.5%) passed our QC. CNV analysis of 6890 subjects detected 176 CNVs in the *PRKN* region. After prioritization, intronic CNVs (*n* = 84) and unlikely pathogenic exonic CNVs (*n* = 20, Table S1) were excluded from further analyses. As a result, 71 participants were identified as carriers of LP‐CNV in *PRKN* (36 SCZ cases, 12 ASD cases, and 23 controls), including one biallelic carrier among the SCZ cases (Figure 2A). Monoallelic carriers of LP‐CNVs in *PRKN* were common in our sample set (70/6890, 1.02%). The frequency was slightly higher in SCZ (35/3014, 1.16%) and ASD (12/1205, 1.00%) than in controls (23/2671, 0.86%). Meanwhile, biallelic carriers of LP‐CNVs in *PRKN* were rare (1/6890). The only biallelic carrier was identified with two LP‐CNVs in *PRKN* (CNV23 and CNV56, Table S1, Figure 2A) and diagnosed with SCZ and EOPD. CNV23 is a duplication in exon 2, while CNV56 is a deletion in exon 6 of *PRKN*, both resulting in a premature stop codon and potential degradation of transcripts by NMD (Figure 2C). Ten other individuals were found to carry similar duplications in exon 2 with uniform length as CNV23 (CNV18–20, CNV24–25, and CNV65–68), but none exhibited Parkinson's symptoms.

### 3.2 Association analysis

With adequate identification of CNVs and a systematic prioritization, we revisited the question regarding whether CNVs in *PRKN* increase the risk of developing NPD, specifically SCZ or ASD. Our initial association analysis conducted on 6889 participants with zero or one LP‐CNV revealed that monoallelic carriers of LP‐CNVs in *PRKN* were not at a higher risk of developing SCZ (OR = 1.35, 95% confidence interval [CI] = 0.80–2.30, *p* = 0.29) or ASD (OR = 1.16, 95% CI = 0.57–2.34, *p* = 0.72) (Table 1). We also conducted a secondary analysis to examine whether monoallelic CNVs spanning specific exon(s) (e.g., exon 2, exons 5–12) of *PRKN* confer an increased risk for NPDs, but no statistically significant results (*p* < 0.05) were found (Table 1). Furthermore, we conducted an association analysis on biallelic carriers and non‐carriers, but due to the limited discovery of only one biallelic carrier (a SCZ case) among all participants, no statistically significant result was found (Table 1).

**TABLE 1:** Association analysis of LP‐CNVs in *PRKN* with risk of SCZ or ASD.

LP‐CNV type	Genotype counts (allelic/non)			ORs (95% CI)			*p*‐value		
	SCZ	ASD	CON	SCZ	ASD	NPD	SCZ	ASD	NPD
Monoallelic									
DEL	28/2985	8/1197	18/2653	1.38 (0.76–2.51)	0.99 (0.43–2.27)	1.27 (0.72–2.24)	0.30	1.00	0.48
DUP	7/3006	4/1201	5/2666	1.24 (0.39–3.92)	1.78 (0.48–6.63)	1.39 (0.48–4.02)	0.78	0.47	0.62
DEL or DUP	35/2978	12/1193	23/2648	1.35 (0.80–2.30)	1.16 (0.57–2.34)	1.30 (0.79–2.14)	0.29	0.72	0.33
E2 DEL^16^	12/3001	2/1203	4/2667	2.67 (0.86–8.28)	1.11 (0.20–6.06)	2.22 (0.73–6.75)	0.09	1.00	0.23
E2 DUP^16^	6/3007	3/1202	5/2666	1.06 (0.32–3.49)	1.33 (0.32–5.58)	1.14 (0.38–3.41)	1.00	0.71	1.00
E2 DEL or DUP^16^	18/2995	5/1200	9/2662	1.78 (0.80–3.96)	1.23 (0.41–3.69)	1.62 (0.75–3.51)	0.18	0.77	0.28
E5‐12 DEL^19^	6/3007	3/1202	3/2668	1.77 (0.44–7.10)	2.22 (0.45–11.01)	1.90 (0.51–7.03)	0.52	0.38	0.39
Region A (E3‐4)^18^	17/2996	5/1200	14/2657	1.08 (0.53–2.19)	0.79 (0.28–2.20)	1.00 (0.51–1.95)	0.86	0.81	1.00
Region B (E5)^18^	3/3010	3/1202	2/2669	1.33 (0.22–7.97)	3.33 (0.56–19.96)	1.90 (0.38–9.43)	1.00	0.18	0.50
Region C (E6‐7)^18^	4/3009	1/1204	2/2669	1.77 (0.32–9.69)	1.11 (0.10–12.24	1.58 (0.31–8.17)	0.69	1.00	0.71
Biallelic									
DEL or DUP	1/3012	0/1205	0/2671	∞	NaN	∞	1.00	1.00	1.00

Abbreviations: ASD, autism spectrum disorder; CI, confidence interval; CON, healthy controls; DEL, deletion; DUP, duplication; E(n): exon (n); LP‐CNVs, likely pathogenic copy number variations; NPD, neuropsychiatric disorder; ORs, odds ratios; SCZ, schizophrenia.

### 3.3 Distribution pattern of CNVs in *PRKN*

Among the LP‐CNVs identified in 6890 Japanese individuals, we observed a clustering toward exons 1–4 encoding Ubl and RING0 domains of Parkin (Figure 2B). Overall, no obvious difference in the distribution of CNVs in *PRKN* was found between NPD cases and controls. CNVs involving exon 2, 3, or 4 accounted for the majority 62/72 (86%) of all LP‐CNVs. Exon‐wise, exon 2 was observed with most of the LP‐CNVs, including 33 CNVs spanning exon 2 and 26 single‐exon CNVs. Notably, 11 of the 13 single‐exon duplications in exon 2 (CNVs 18–20, 23–26, and 65–68) were uniform in width (~300 kb), accounting for 85% of the duplications in exon 2 of *PRKN*. This finding was consistent with a recent large‐scale study in the European population showing that CNVs in exons 2–4 of *PRKN* are common and that duplications found in exon 2 were uniform in size.^25^ Intriguingly, the duplications identified in our sample set of the Japanese population were larger (~300 kb) than those identified in that study (~200 kb). Given the high‐resolution of CNV detection methods in both our study (NimbleGen or Agilent) and the previous study (NeuroX^26^ or NeuroChip^27^), the 100‐kb difference in length suggested that the two duplications were likely distinct variants. However, whether the 300‐kb duplication discovered in our study is population‐specific requires further investigation.

## 4 DISCUSSION

In the present study, we identified a greater number of LP‐CNVs in *PRKN* in NPD cases compared with previous studies.^16^, ^18^, ^19^ However, after systematic prioritization and examination, we found that monoallelic carriers of LP‐CNVs in *PRKN* were not at a higher risk of developing SCZ or ASD (Table 1), which contradicts previous findings that monoallelic CNVs may confer an increased risk of developing NPDs.^16^, ^17^, ^18^, ^19^ Yin et al.^18^ reported a higher frequency of *PRKN* CNVs in ASD cases compared with controls (0.94% vs. 0.14%, respectively; *p* = 0.014) by screening 1428 participants (335 ASD cases and 1093 controls) for CNVs in *PRKN* and genotyping on designated regions for replication in 301 ASD cases and 301 controls. Jarick et al.^16^ reported a similar result in ADHD cases (1.04% vs. 0.13%, respectively; *p* = 0.043) in the replication samples of 386 ADHD cases and 781 controls. However, the frequency of CNVs in *PRKN* in their control samples was much lower than that in our study (0.86%) and other large‐scale studies (0.55–0.95%^25^, ^28^, ^29^). This omission of CNVs may have been caused by limitations in the detection methods employed, and may have influenced their results. Moreover, we were unable to replicate the excess of CNVs among NPD cases in the risk regions suggested by their studies (i.e., exon 2, exons 3–4, exon 5, and exons 6–7) (Table 1). Instead, our observations were more consistent with the results of Conceição et al.,^19^ who reported that CNVs in exons 1–4 of *PRKN*, which encode the Ubl and RING0 domains of Parkin, were frequently observed in both controls and NPD cases. We found that LP‐CNVs in exons 2–4 were the most common, whereas LP‐CNVs in exons 5–12 were infrequent. Notably, exons 5–12 of *PRKN* encode the RBR domain, which is the main functional domain of Parkin, and an excess of CNVs in this region has been reported in NPD cases.^19^ However, our association analysis did not yield any statistically significant evidence to suggest that monoallelic LP‐CNVs in exons 5–12 of *PRKN* are associated with an increased risk of NPDs (Table 1). Overall, while the possibility of monoallelic CNVs in *PRKN* conferring a higher risk of SCZ or ASD remains, as low‐penetrance from monoallelic CNVs in *PRKN* for Parkinson's disease (PD) has been suggested,^30^ our data showed that any potential risk should be limited.

Only one carrier of biallelic pathogenic CNVs in *PRKN* was identified among the NPD cases in our study (none in the control group); however, due to limited number of cases, statistical significance was not achieved. In fact, the low prevalence of biallelic CNVs in *PRKN* (1/6890) makes achieving statistical significance challenging. To achieve a statistical power of 0.8, a minimum of 200 000 participants is estimated to be required, even when combining other pathogenic variants such as disruptive single‐nucleotide variations (SNVs). Future studies will require more complex study designs to investigate the role of biallelic pathogenic variants in *PRKN* in NPDs, rather than relying solely on large sample sizes in a one‐stage genetic association study.

Nevertheless, the discovery of this biallelic carrier is valuable, as this carrier was also diagnosed with EOPD, a recessive form of PD commonly caused by pathogenic variants in *PRKN*.^31^ This diagnosis supports the pathogenicity of the two CNVs (CNV23 and CNV56) carried by the individual, suggesting that they may lead to the functional loss of both copies of *PRKN* in a compound heterozygous manner. This finding is significant because 10 other monoallelic carriers of the same pathogenic CNVs in exon 2 did not exhibit PD symptoms, further highlighting the lack of effect of monoallelic pathogenic CNVs in *PRKN* in the absence of a second hit.

Here, we reported the frequency and distribution of LP‐CNVs in *PRKN* in a Japanese population of 6890 participants by systematically screening for CNVs using high‐resolution arrays. We found that monoallelic carriers of LP‐CNVs in *PRKN* were relatively common (1.02%), whereas biallelic carriers were rare (1/6890). The distribution of CNVs in NPD cases and controls showed no obvious differences, and the clustering toward exons 2–4 was similar to that observed in other populations.^25^ Notably, we identified 300‐kb duplications in exon 2, which were larger than those previously reported in the European population,^25^ but were also uniform in length and likely had identical consequences, as they both involved only exon 2. Whether this 300‐kb duplication is population‐specific requires further investigation.

To our knowledge, our case–control study is the first to systematically examine the association of CNVs in *PRKN* with SCZ since the original discovery,^9^ and the largest to examine the association of CNVs in *PRKN* with NPD.^16^, ^18^, ^19^ Therefore, we have provided a better perspective for interpreting CNVs in *PRKN* in patients with NPDs by highlighting the importance of reassessing results from previous genetic studies and reporting negative results, which was also suggested by recent large‐scale studies on *PRKN*.^25^, ^28^, ^32^

This study does have a few limitations worth mentioning. First, while aCGH is a sensitive and reliable method for detecting CNVs, it cannot identify SNVs. Therefore, it is possible that there may be biallelic carriers with another pathogenic SNV that were detected. However, because pathogenic SNVs in *PRKN* are rare,^25^ this limitation is unlikely to affect the main conclusion of the present study. Second, CNV detection in this study was performed using two platforms, NimbleGen and Agilent. While some may question the validity of this approach, our previous research has demonstrated that the CNV detection resolution of these two platforms is comparable under the settings used in this study.^33^

In conclusion, the findings of the present study indicate that monoallelic CNVs in *PRKN* do not confer a significant risk for SCZ or ASD. However, further studies are warranted to investigate the association between biallelic CNVs in *PRKN* and NPDs. We also found that CNVs in *PRKN* were relatively common among the Japanese population and that their distribution was no different from that of other populations.

## AUTHOR CONTRIBUTIONS

T.L. and I.K. designed the study. I.K., B.A., and T.L. performed the aCGH and/or validation experiments. T.L. and I.K. analyzed the data. I.K., T.O., Hiroki K., Hidekazu K., T.I., Y.N., Y.T., M.Y., R.K., Y.F., Hirotaka K., S.N., K.K., Tsukasa S., S.Y., T.M., N.I., Masashi I., R.H., Masanari I., M.A., K.O., Toshiyuki S., Y.W., J.E., T.T., M.S., H.Y., and N.O. recruited the participants and/or collected DNA samples or phenotype data. T.L. wrote the first draft of the manuscript, and the other authors commented on and refined the subsequent versions. All authors carefully read the manuscript and approved the final version for submission.

## CONFLICT OF INTEREST STATEMENT

I.K. has received research grants from the SENSHIN Medical Research Foundation and Uehara Memorial Foundation. M.I. has received research grants from AMED. N.O. has received research support or speakers' honoraria from, or has served as a consultant to, Sumitomo Dainippon, Eisai, Otsuka, KAITEKI, Mitsubishi Tanabe, Shionogi, Eli Lilly, Mochida, DAIICHI SANKYO, Nihon Medi‐Physics, Takeda, Meiji Seika Pharma, EA Pharma, Pfizer, MSD, Lundbeck Japan, and Taisho Pharma, outside the submitted work. All other authors report no biomedical financial interests or potential conflicts of interest.

## ETHICS STATEMENT

This study was approved by the ethics committee of Nagoya University Graduate School of Medicine; Genomic study for brain and mental disorders and effectiveness and side effect of pharmacological treatment (2010–1033). This study complied with all the provisions of the Declaration of Helsinki.

Informed consent: Written informed consent was obtained from all the participants.

Registry and the registration No. of the study: N/A.

Animal studies: N/A.

## Supporting information


Table S1.

## Data Availability

The data that support the findings of this study are available in the supporting information of this article. Due to privacy and ethical considerations, the raw CNV data containing personal information cannot be publicly shared. However, to promote transparency and facilitate reproducibility, a de‐identified and aggregated table of identified exonic CNVs in *PRKN*, without any personally identifiable information, is included as a supplemental file.
